# Challenges and limitations of anal cancer and precancer screening among people with HIV in a real-world setting

**DOI:** 10.1007/s15010-026-02748-4

**Published:** 2026-02-26

**Authors:** V. Menzel, E. Gruener, J. Neuf, S. Sammet, S. Esser, J. Roider, U. Seybold

**Affiliations:** 1 Division of Infectious Diseases, Department of Medicine IV, LMU University Hospital, LMU Munich, Pettenkoferstr. 8a, 80336 Munich, Germany; 2 Department of Dermatology and Venereology, University Hospital Essen, University Duisburg-Essen, Essen, Germany; 3 German Centre for Infection Research (DZIF), Partner Site, Munich, Germany

**Keywords:** HIV, Cancer screening, Anal cancer, Anal cytology, Anal intraepithelial neoplasia, High resolution anoscopy

## Abstract

**Purpose:**

Anal carcinoma (AC) incidence in people with HIV (PWH) is increasing. Guideline-recommended anal-cytology screening facilitates early treatment of precancerous lesions. Data on real-world adherence to screening- and follow-up (F/U)-recommendations remain limited.

**Methods:**

We analyzed routine anal-cytology screening in PWH at a German university hospital-clinic (2017–2023), including frequency of findings, F/U, and final diagnoses.

**Results:**

The study-population included 434 persons with 936 anal-cytology results, representing 25% of person-time of F/U at our clinic. Median age was 55 years, female/male ratio was 5/429, 310 (71%) were MSM. Median CD4-nadir was 242/µl, in 97% viral load was suppressed, 47% were currently smoking.

Of 936 swab-results, 882 (94%) were evaluable; 201 (21%) showed abnormalities. Among these, 60 (30%) were ASC-US, 77 (38%) LSIL, 12 (6%) ASC-H, and 52 (26%) HSIL. High-resolution-anoscopy (HRA), recommended for the 64 ASC-H/HSIL-cases, was performed in 7 (11%), identifying 1 AIN2. In 46/64 (72%) proctoscopy identified 1 AC and 3 cases each of AIN2/AIN3. Eleven persons (17%) refused F/U. Thus, 8/64 (12.5%) suspicious cytology findings were histopathologically confirmed. During the study period, 4 cases of AC were diagnosed in unscreened persons at our clinic.

**Conclusion:**

Routine anal-cytology screening in an unsystematically selected cohort of 434 PWH contributing 1,383 person-years of F/U identified 7 cases of high-grade precancerous lesions and 1 asymptomatic AC. Important challenges included low uptake, limited specificity of cytology, inconsistent adherence to F/U recommendations, and insufficient availability of HRA. Improved communication of HIV-care-providers with all involved parties will be a key requirement for improving efficiency and outcomes.

## Introduction

The incidence of anal carcinoma (AC) among people with HIV (PWH) is increasing [1]. Approximately 90% of anal cancers are caused by persistent human papillomavirus (HPV) infection [2, 3], most frequently high-risk HPV (HR-HPV). HR-HPV infections can lead to dysplastic changes in the anal canal, which can develop into AC over time if left untreated [4, 5]. HR-HPV infection is more prevalent in PWH, especially in men who have sex with men (MSM) or transgender women [6]. Further risk factors for anal HPV-associated changes include older age, a low CD4 + T-lymphocyte cell count nadir, the number of sexual partners, and smoking [1, 7–9].

The recently updated German-Austrian screening guideline recommends cytology screening for PWH and other persons at increased risk for AC to facilitate early detection and treatment of precancerous lesions [10]. The results of the randomized ANCHOR study among 4,446 PWH aged 35 years and older who had a precursor lesion support this approach: here, treatment of anal dysplasia statistically significantly reduced the incidence of anal cancer compared to the observation group [11]. Analysis of the Dutch nationwide ATHENA cohort including 28,175 PWH demonstrated a significantly lower mortality among men diagnosed with AC when participating in a screening program [12].

However, there may be challenges to the effectiveness of such programs, as demonstrated by a recent five-year evaluation of an anal cancer screening program implemented at two centers in the US, which reported limitations in adherence along the care cascade [13]. Less than ideal adherence to screening intervals and follow-up procedures could negatively affect the effectiveness of a cancer screening program compared to a rigorous clinical trial setting and could occur at any step of the program. This includes the care providers’ recommendation to undergo anal-cytology screening, the acceptance by the individual, the collection of a sufficiently evaluable cytology specimen, the timely discussion of the result with the individual, the timely implementation of recommended follow-up procedures, and finally the continuation of follow-up screening examinations.

Due to the still limited data on the implementation of anal-cytology screening in real-world settings especially in Europe this study therefore aimed to quantify uptake and outcomes of the anal cancer screening program for PWH at our institution. We specifically aimed to characterize limitations of anal cytology based screening, including adherence to recommendations and proportion of follow-up at different steps of the process. Addressing these limitations will be necessary to improve program efficiency and patient outcomes.

## Methods

This observational, longitudinal study was approved by the Institutional Review Board of the Faculty of Medicine at Ludwig-Maximilians-University Munich (LMU; project number 23–0260). PWH attending the outpatient clinic at the Division of Infectious Diseases, Department of Medicine IV at LMU Hospital, with at least one anal-cytology screening specimen documented between January 2017 and March 2023 were included in a retrospective analysis of anonymized data collected during routine visits. This clinic provides comprehensive HIV care for about 850 PWH, including antiretroviral therapy, routine laboratory monitoring, management of comorbidities, and preventive health services. The latter include cytology-based anal cancer screening in cooperation with a specialized cytology laboratory and several proctology experts both within and outside the university hospital. The clinic plays a central role in HIV care within the regional healthcare system by integrating anal cancer and precancer screening into routine clinical follow-up. While multiple other options for HIV care are available in Munich, access to proctologists who specialize in anal cancer and precancer screening in the context of HIV is limited, which makes referral and follow-ups logistically challenging. As part of routine clinical care, anal cancer screening based on cytology smear tests was offered to all PWH at the clinic on a yearly basis as suggested by the previous version of the guideline [14].

With individuals lying down in the left lateral position with hips flexed, a cytology brush (medex Cytobrush, Medesign, Dietramszell, Germany) moistened with NaCl 0.9% was inserted into the anal canal and then rotated with gentle pressure while slowly withdrawing it. The brush was then rolled out on a glass microscope slide before application of an alcohol-based fixing spray (Cyto-Spray Fixative, Medesign, Dietramszell, Germany). Cytologic evaluation was performed by specifically trained cytopathologists at Amedes MVZ for Gynaecology and Pathology laboratory, Munich, Germany, according to the Bethesda System [15]. Cytology results, number of swabs per patient, adherence to recommended follow-up, and final disposition (i.e., no abnormality detected by anal-cytology or diagnostic outcome of proctology referral) were analyzed. Therapeutic interventions were performed outside of our clinic and thus are not addressed by this analysis of our screening program. Baseline characteristics were compared between individuals with at least one follow-up (F/U) cytology or proctology referral vs only one swab without any further F/U. Correlation between suspicious cytology and histopathology, as well as HRA accessibility were evaluated. Incidence of AC and premalignant changes were assessed and compared to PWH attending the clinic but not undergoing cytology screening during the same period.

Statistical analyses were performed with Microsoft Excel and GraphPad Prism version 10.1.2. Chi-Square- and Mann–Whitney-tests were used for two-group comparisons where applicable. P-values < 0.05 were considered statistically significant.

## Results

### Study population and distribution of anal cytology screening swabs

The study-population includes 434 persons contributing a total of 936 anal swab results over the 75-month study period with 1,383 person-years (py) of F/U in the screening program. During the same 75-month period, individuals at the clinic not receiving anal cytology screening contributed 4,078 py of F/U, thus uptake was 25.3%. Median age was 55 years (IQR: 47–61), female/male ratio was 5/429, 310 (71%) were MSM. Median CD4-nadir was 242/µl (IQR: 125–368), 97% had a viral load < 50 cp/ml, 47% were currently smoking. Detailed baseline characteristics are shown in Table 1 (Table 1).

**Table 1:** Baseline characteristics of 434 people with HIV undergoing anal cytology-based screening between 2017 and 2023

Variables^a^	Total (N = 434)	No follow-up^b^ (N = 159)	Follow-up^c^ (N = 275)	
	median (IQR)	median (IQR)	median (IQR)	p-value
Age [years]	55 (47–61)	51 (44–59)	56 (48–62)	**0.001**
Years on cART^d^	8 (4–16)	7 (2–14)	9 (5–16)	0.48
Bodyweight [kg]	80 (71–91)	82 (72–91)	80 (71–91)	0.65
CD4 cell count nadir [/µl]	242 (125–368)	248 (136–386)	236 (122–357)	0.27
**CD4 cell count**				
absolute count/µl	582 (425–728)	565 (412–741)	588 (432–716)	0.57
% of lymphocytes	33 (27–40)	34 (27–41)	33 (27–39)	0.71
**CD8 cell count**				
absolute count/µl	663 (488–861)	642 (478–851)	683 (488–874)	0.48
% of lymphocytes	37 (31–46)	37 (31–47)	37 (31–45)	0.51
**Gender**				0.44
cis-male	429 (98.8)	158 (99.4)	271 (98.5)	
cis-female	5 (1.2)	1 (0.6)	4 (1.5)	
other	0 (0.0)	0 (0.0)	0 (0.0)	
**HIV transmission risk category**				0.92
MSM^e^	310 (71.4)	112 (70.4)	198 (72.0)	
heterosexual	54 (12.5)	21 (13.2)	33 (12.0)	
Origin from high-prevalence country^f^	51 (11.8)	20 (12.6)	31 (11.3)	
other^g^	19 (4.3)	6 (3.8)	13 (4.7)	
**CDC clinical category (1993 classification)**				0.22
A^h^	213 (49.1)	85 (53.5)	128 (46.5)	
B (history of HIV-associated illness)	110 (25.3)	33 (20.8)	77 (28.0)	
C (history of AIDS-defining illness)	111 (25.6)	41 (25.8)	70 (25.5)	
**CDC immunological category (1993 classification)**				0.27
1 (CD4-nadir ≥ 500 cells/µl)	52 (12.0)	23 (14.5)	29 (10.5)	
2 (CD4-nadir ≥ 200–499 cells/µl)	188 (43.3)	72 (45.3)	116 (42.2)	
3 (CD4-nadir < 200 cells/µl)	194 (44.7)	64 (40.3)	130 (47.3)	
**Plasma viral load**				0.11
≤ 50 copies/ml	420 (96.8)	151 (95.0)	269 (97.8)	
> 50 copies/ml	14 (3.2)	8 (5.0)	6 (2.2)	
**cART**^**d**^ **regimen**				**< 0.0001**
3-drug combination	270 (62.2)	129 (81.1)	141 (51.3)	
2-drug combination	158 (36.4)	29 (18.2)	129 (46.9)	
≥ 4-drug combination	4 (0.9)	1 (0.6)	3 (1.1)	
monotherapy	2 (0.5)	0 (0.0)	2 (0.7)	
**Smoking status**				0.30
current	203 (46.8)	80 (50.3)	123 (44.7)	
former	84 (19.3)	25 (15.7)	59 (21.5)	
never	147 (33.9)	54 (34.0)	93 (33.8)	

^a^All variables at time of first anal cytology swab;^b^No follow-up: n = 159 people with HIV with only a single anal-cytology swab;^c^Follow-up: n = 275 people with HIV with follow-up either by additional anal-cytology swabs or referral to a proctologist;^d^cART: combination antiretroviral therapy;^e^MSM: men who have sex with men;^f^origin from countries where HIV is endemic and predominantly transmitted heterosexually, with an HIV prevalence greater than 1% in the general adult population (aged 15–49 years), based on the most recent UNAIDS reports.^g^other includes blood transfusion, intravenous drug use, or unknown.^h^asymptomatic HIV infection, persistent generalized lymphadenopathy (PGL), or acute HIV illness

**Fig. 1:**
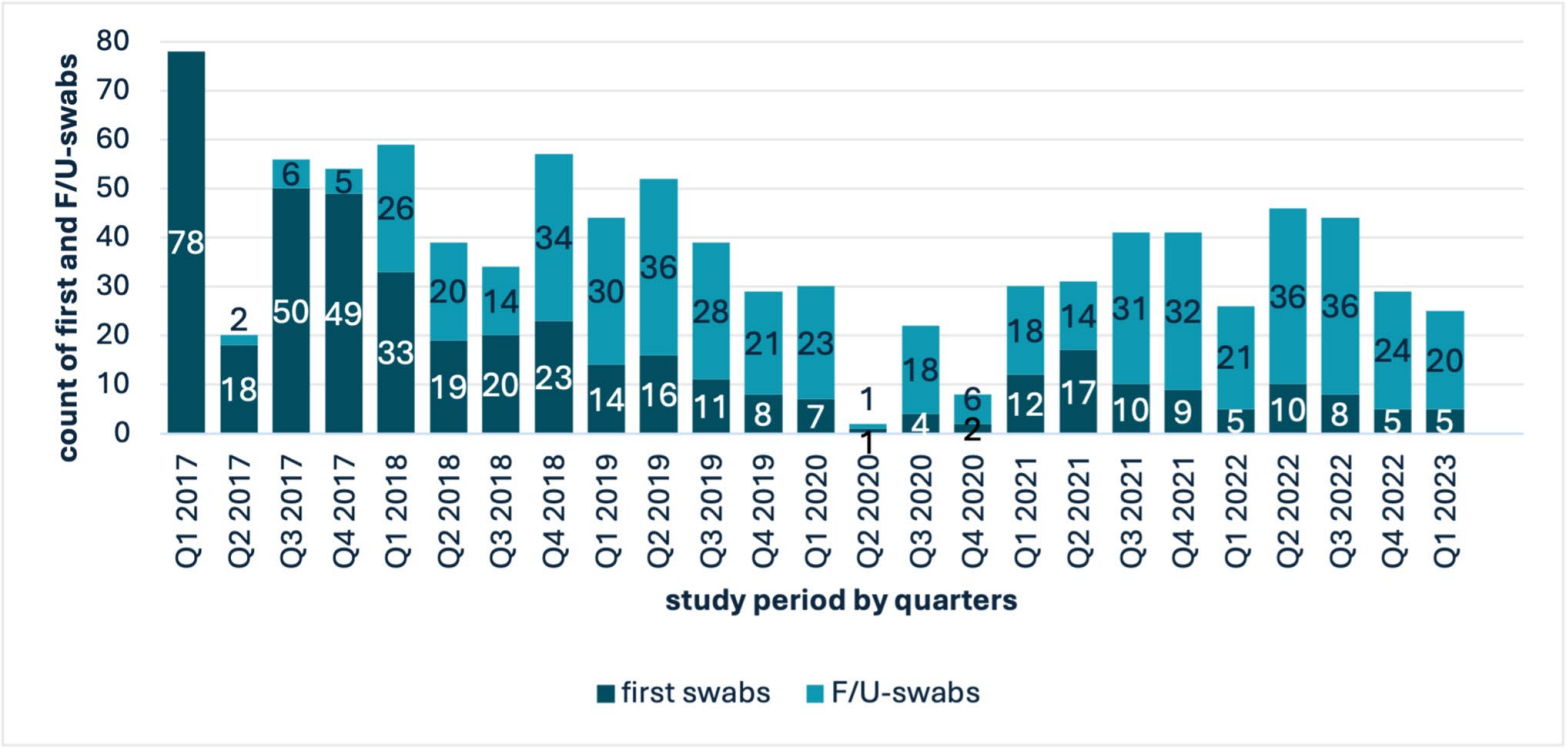
Distribution of first and follow-up anal cytology swabs during the study period by quarters First swabs (dark blue bar; n = 434); follow-up swabs (F/U-swabs, light blue bar; n = 502)

The number of swabs per individual ranged from 1 to 6. As to be expected, most of the first swabs were collected during the early years of the study period in 2017 and 2018, while collection of F/U swabs was relatively constant since 2018. Few swabs overall were collected during 2020, when restrictions due to the COVID-19 pandemic were in place (Fig. 1).

At the time of the study (2017–2023) F/U-swabs were recommended at yearly intervals [14]. Despite this recommendation, we identified 159/434 individuals (37%) who after an initial screening never received any F/U by either repeat anal cytology or proctology referral. In this group, the proportion of high-grade intraepithelial lesions or atypical squamous cells cannot exclude high-grade squamous intraepithelial lesions (HSIL/ASC-H) was significantly higher compared to participants who did receive F/U (8.8% vs 3.6%; p = 0.02), while the proportion of low-grade intraepithelial lesions (LSIL) was significantly lower (3.8% vs 9.1%; p = 0.04) (Table 2). Characteristics associated with not receiving F/U after an initial cytology result included younger age (p = 0.001) and a 3-drug as opposed to a 2-drug anti-retroviral therapy regimen (*p* < 0.001, Table 1).

**Table 2:** Initial anal-cytology screening results among 434 people with HIV, 2017—2023

Initial anal-cytology result	Total (N = 434)	No follow-up^a^ (N = 159)	Follow-up^b^ (N = 275)	p-value
	n (%)	n (%)	n (%)	
		159 (100.0)	275 (100.0)	
Not evaluable	28 (6.5)	9 (5.7)	19 (6.9)	0.61
No abnormality detected	324 (74.7)	123 (77.4)	201 (73.1)	0.32
LSIL^c^	31 (7.1)	6 (3.8)	25 (9.1)	**0.04**
ASC-US^d^	27 (6.2)	7 (4.4)	20 (7.3)	0.23
ASC-H^e^ or HSIL^f^	24 (5.5)	14 (8.8)	10 (3.6)	**0.02**

^a^ No follow-up: n = 159 people with HIV with only a single anal-cytology swab; ^b^ Follow-up: n = 275 people with HIV with follow-up either by additional anal-cytology swabs or referral to a proctologist; ^c^ LSIL: low-grade intraepithelial lesion; ^d^ ASC-US: atypical squamous cells of undetermined significance; ^e^ ASC-H: atypical squamous cells cannot exclude high-grade squamous intraepithelial lesion, ^f^ HSIL: high-grade intraepithelial lesion

### Screening results

**Fig. 2:**
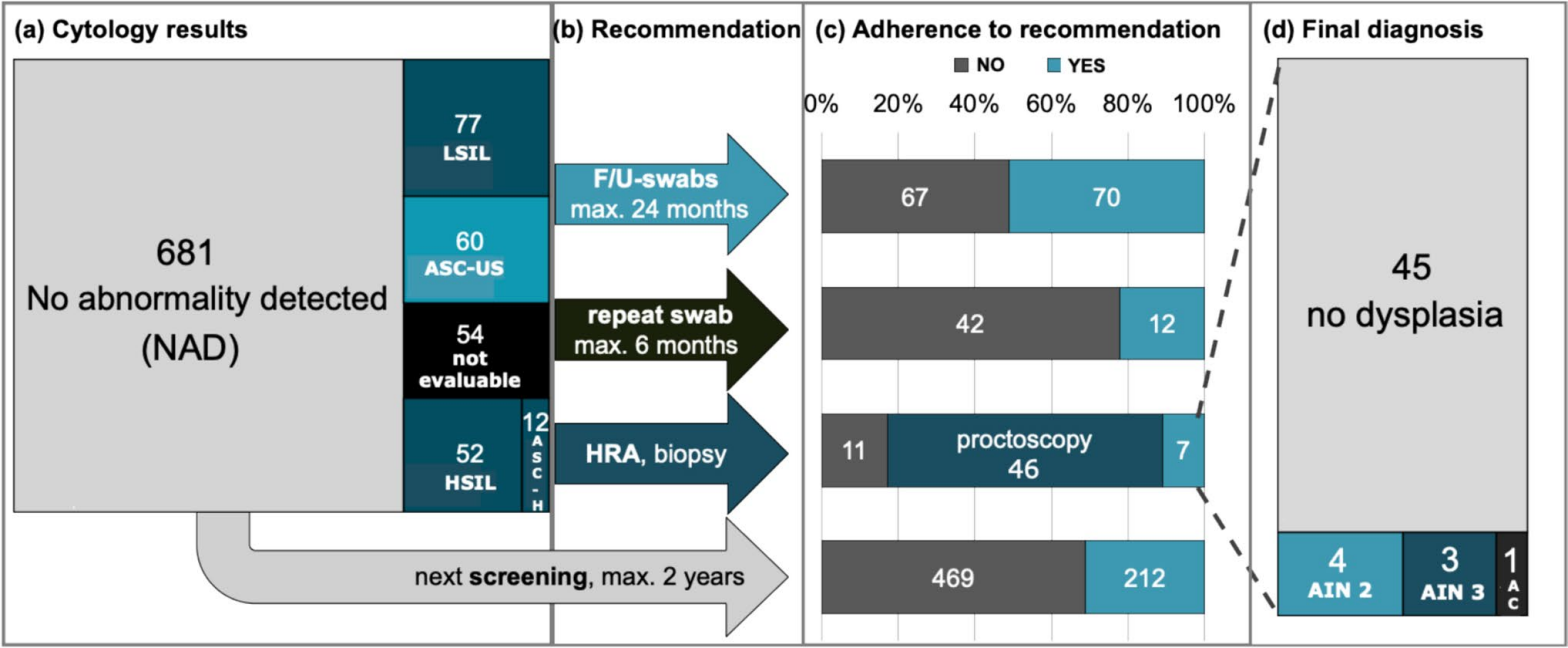
Anal cytology results, recommended follow-up, adherence, and final diagnosis among 434 people with HIV, 2017 – 2023 **a** Anal cytology screening results from routine clinical samples (n = 936) collected during the study period from 2017–2023. No abnormality detected (NAD; n = 681), low-grade intraepithelial lesion (LSIL; n = 77), atypical squamous cells of undetermined significance (ASC-US; n = 60), atypical squamous cells cannot exclude high-grade squamous intraepithelial lesion (ASC-H; n = 12), and high-grade intraepithelial lesion (HSIL; n = 52). **b** Specific recommendations according to screening result: LSIL/ASC-US: repeat swab within 12 months; not evaluable: repeat swab within 6 months; ASC-H/HSIL: referral to HRA and biopsy; NAD: repeat swab within two years. **c** Adherence to specific recommendation among study cohort: number of persons following the recommendation given in light blue area, number not adherent to recommendation in dark grey; recommendation for HSIL/ASC-H: despite recommendation for HRA, proctoscopy was performed in 46 cases (dark blue). **d** Final, histopathologically confirmed diagnosis: AIN2 (n = 4), AIN3 (n = 3), AC (n = 1); no evidence for dysplasia (n = 45), among these inflammatory lesions (n = 18) including hemorrhoids (n = 9), condyloma (n = 5), inflammatory plaques (n = 2), minimal rectitis (n = 1), anal skin tags (n = 1)

Out of 936 swabs, 54 (5.8%) were not evaluable. Among 882 evaluable swabs, 681 (77.2%) were classified as no abnormality detected (NAD), 77 (8.7%) as LSIL, 60 (6.8%) as atypical squamous cells of undetermined significance (ASC-US), 12 (1.4%) as ASC-H, and 52 (5.9%) as HSIL (Fig. 2a).

Depending on the result, specific F/U was recommended (Fig. 2b), however adherence was incomplete. Following NAD, a F/U-swab was collected within the currently recommended [10] interval of two years in 212/681 cases (31%). After not evaluable results, 12/54 cases (22%) had a timely repeat swab within 3–6 months. Following LSIL and ASC-US, repeat swabs within 6–12 months were collected in 70/137 cases (51%). For 64 ASC-H/HSIL cases proctology referral for HRA was recommended, but 11 individuals (17%) refused proctology referral, 46 (72%) underwent proctoscopy instead, and HRA was performed in only 7 (11%) (Fig. 2c). The median time between cytology and HRA or proctoscopy was 6 months (IQR 3–12).

Among 7 participants undergoing the gold-standard of HRA, biopsy was performed in 4, identifying one case of anal intraepithelial neoplasia (AIN)2 (14%). Among the 6 instances with no evidence of dysplasia, an anal skin tag and hemorrhoids were diagnosed in 1 and 2 cases, respectively. Among 46 participants undergoing proctoscopy, biopsy was performed in 10, identifying 3 cases each of AIN2 (7%) and AIN3 (7%), as well as 1 early-stage AC (2%). Proctoscopy revealed no evidence of dysplasia in 39/46 cases (85%), including 7 cases of hemorrhoids, 1 minimal rectitis, 5 with condyloma, and 2 with inflammatory plaques.

Crude AC-incidence was lower in the screened group with one AC over 1,383 py (0.7/1000 py) compared to the unscreened group with 4 cases over 4,078 py (1.0/1000 py), including one with an initially unsuspicious swab who was lost to F/U and then diagnosed with AC three years later. Incidence of AIN2 and AIN3 in the screened group were 2.9/1000 py and 2.2/1000 py, respectively.

## Discussion

In this study, we assessed an anal-cytology screening program for people with HIV in the real-world setting of a German university hospital outpatient clinic. Collection of 936 cytology swabs from 434 individuals contributing 1,383 py of follow-up over a 75 month-period resulted in the early detection of one AC and 7 high-grade premalignant lesions.

As anal-cytology screening is minimally invasive, inexpensive and relatively easy to perform [16], it was universally offered to all individuals with HIV attending the clinic. Acceptance in our clinic was just over 25%, which compares favorably to 15% reported among US veterans [17] but is significantly lower than the recently reported 50.7% at two academic HIV clinics in the US [13]. Among the 936 anal-cytology swabs collected, 201 results (21%) required further work-up, including 64 instances (6.8%) of HSIL or ASC-H for which HRA and biopsy was recommended. Eventually, 7 cases of AIN2 and AIN3 and 1 early-stage AC were confirmed by histopathology. The early detection of HSIL and their subsequent evaluation and timely treatment may have prevented progression to AC [18, 19], thus the screening efforts likely avoided more complicated treatment in 7 persons [20], and ultimately might have contributed to increased survival [12].

Crude incidence of AC in our screening cohort was 0.7/1,000 person-years, comparing favorably to the crude incidence of 1.0/1,000 person-years among the persons not undergoing routine screening at our clinic during the same period. Even though the low absolute numbers and the relevant potential for immortal-time bias do not allow for statistically meaningful comparisons, a lower rate of AC among persons in screening has also been reported by others [21]. Considering the additional incidence of AIN2/AIN3 at 5.1/1,000 person-years among the persons screened, the screening offer may have been accepted by persons at higher perceived risk which underlines the overall justification of the screening program.

**Fig. 3:**
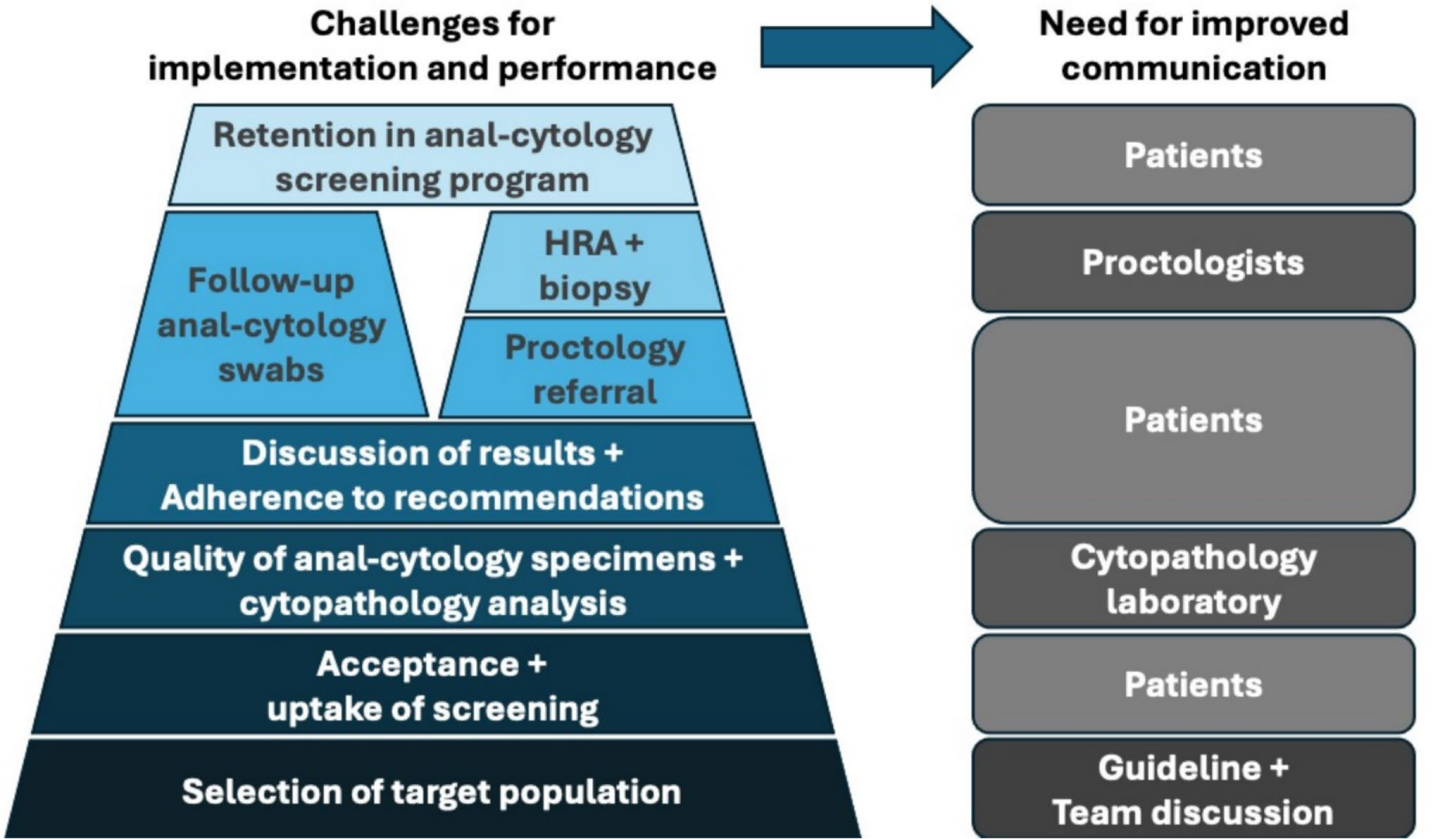
Challenges of an anal-cytology screening program and communication requirements for HIV care providers

Our analysis also identified several challenges and limitations affecting both the efficiency of the screening program and the timeliness and quality of patient care (Fig. 3). Most importantly, about 75% of our outpatients did not undergo anal cytology screening at all. Additional analyses by our group show that this was primarily due to the providers not specifically recommending it during the clinic visits [22]. Individuals not in the screening program were more often female and, despite their known increased risk, significantly older [22]. Specific efforts are necessary to overcome this obvious bias, likely prevalent both among HIV care providers and people with HIV.

Once in screening, protocol adherence was mostly insufficient, especially with respect to recommended intervals between swabs. This also was true for one person diagnosed with AC following an unsuspicious swab three years earlier but no further F/U. Alarmingly, individuals who only received one anal-cytology swab but never had a F/U had a significantly higher proportion of HSIL/ASC-H. Also, younger age was significantly associated with a lower proportion of F/U. Apparently, additional communication efforts are necessary to better motivate this at-risk group to continue with anal-cytology screening and especially to accept proctologic evaluation of suspicious results.

Among 64 persons with high-grade suspicious cytology results, 11 (17%) declined any further evaluation, and among 53 accepting proctology-referral only 7 (13%) actually underwent HRA. Despite being the international gold-standard for F/U of suspicious cytology results including HSIL and ASC-H, availability remains very limited also in high-resource settings such as Germany and Austria [10], other Western European countries, Australia [18, 19], and the US [23]. HRA requires significant training and costly equipment [18] and thus was only performed by few specifically trained and highly experienced providers accessible for our patients. It is not adequately reimbursed in the German medical system, therefore more widespread availability is not expected in the near future. Standard proctoscopy without the use of optical magnification, performed in the majority of cases with HSIL/ASC-H in our study, is a guideline-accepted alternative. However, its lower sensitivity for anal dysplasia, even when using acetic acid or Lugol’s solution staining, at the same level of procedural burden will have to be discussed with patients [10].

In addition, F/U information and final diagnoses after further proctologic work-up were difficult and in some cases impossible to obtain for the HIV care providers. Both this difficulty and the key importance of close interdisciplinary communication and collaboration between HIV care providers and proctologists for successful screening, valid and prompt diagnosis by HRA, adequate treatment, and F/U of HSIL and AC [18–20] were important insights during our investigation resulting in increased efforts to build a stronger provider network.

The overall workload for identification of an ultimately small number of confirmed diagnoses was high and the predictive value of cytology for (pre)malignant lesions was low. Liquid based cytology, additional p16 staining, and HPV testing could have increased specificity. However, these tests are not offered on a routine basis in Germany due to reimbursement issues. Only 8/53 (15%) of high-grade suspicious cytology results undergoing further proctologic evaluation were confirmed as (pre)malignant lesions by histopathology, suggesting low specificity of cytology results. In 27 (42%) no abnormality was detected, while in 18 (34%) inflammatory lesions were identified. Additional investigations of ano-mucosal immunology in a subgroup of study participants revealed a predominantly proinflammatory profile in individuals with abnormal cytology [24]. This correlates well with the significantly higher proportion of inflammatory compared to (pre)malignant lesions following up high-grade anal-cytology results.

With a frequency of suspicious findings of half or even less at every step of the process, including cytology, HRA/proctoscopy, and histology, compared to others [13, 25], screening was less efficient in our asymptomatic and thus not specifically highest-risk population, certainly also due to lower disease frequency [26]. A more specific focus on high-risk groups such as MSM living with HIV [16] and older persons is among the recommendations by the current German-Austrian guideline [10]. This will likely improve the predictive value of cytology. However, with only individuals included who actually accepted the screening, our study population already represents a convenience sample with at least a certain degree of selection bias towards a higher risk. Comparing our study population to the target populations suggested by the current guideline, 78.3% were 45 years of age or older, 67.5% were MSM aged 35 years or older, and 41.0% had a CD4 + count nadir below 200/μl. Despite the universal screening offer based on the previous guideline [14], only 14/434 (3.2%) among study participants did not meet any of the criteria listed by the current guideline [10].

Digital anorectal examination (DARE) is also suggested by the guideline as an additional mode of examination before cytology screening. This was not routinely performed in our setting and could potentially improve identification of patients for HRA referral. With the recommendation for HRA also for LSIL- and ASC-US findings on cytology, sensitivity of early detection of (pre)malignant lesions should improve and demand for HRA will certainly increase [10]. The now extended 2-year anal-cytology screening intervals following NAD-findings on the other hand may facilitate guideline implementation. However, with just 31.1% adherence among our study population even this recommendation may be a challenge, also for screening programs elsewhere.

In addition to adequate protocol adherence, high quality diagnostic performance, and adequate access to HRA are prerequisites for effective real-world implementation of anal-cytology screening [17, 25]. All will have to be addressed in order to improve efficiency of AC screening in our setting (Fig. 3).

Our analysis has limitations that need to be discussed. The vast majority of participants are men, with only 5/434 (1.2%) being women and no trans-persons included. This is not representative of the community served by our clinic and thus in contrast to current guidelines [10], but clearly reflects the subconscious and potentially conscious biases preventing us and potentially others from delivering optimal care [22]. Our study is monocentric and includes a limited number of individuals. However, we were still able to include about twice as many individuals compared to a recent study analyzing anal-cytology screening at 2 US centers [13]. Inclusion did not address a specifically selected highest-risk population. However, with 97% qualifying for current guideline-recommended [10] screening this situation likely represents a real-life setting comparable to at least other university hospital clinics in Germany and Western Europe, where both similar baseline conditions and similar challenges may be expected. Liquid based cytology, p16 staining, and HPV testing all could have improved test characteristics, but are not routinely reimbursed in the Germany healthcare system. While the use of conventional cytology applies to other German institutions as well, it limits generalizability of our findings for environments with access to other testing modalities. Finally, our analysis addressed the implementation of screening diagnostics, thus the program’s impact on specific therapeutic interventions was beyond the scope of our study.

There are also specific strengths of our study that are worth mentioning. The detailed characterization of our study population allowed for the comparison to current screening recommendations, helping to assess reasons for apparent lack of specificity and predictive value. Most importantly, with an analysis starting at the screening recommendation and then following up all participants through their final disposition, we were able to identify and quantify important challenges highlighting the need for improved communication. These were found at different stages of the screening process and involve HIV care providers at the clinic, persons in the screening program, cytopathology diagnostics, as well as HRA and proctology follow-up. All of them will now have to be specifically addressed in order to improve future quality of care (Fig. 3). Doing so will primarily require improved and intensified communication of HIV care providers with all parties involved in the process.

## Conclusion

Early detection of precancerous and cancerous anal lesions can reduce anal cancer rates through timely intervention. Our analysis identifies limitations of an anal-cytology screening for people with HIV in a real-world setting. While the updated German-Austrian guideline provides a clear framework for more efficient anal-cytology screening [10], a number of challenges remain, including the need for improved access to HRA. Addressing these challenges and limitations as well as continuous reevaluation of screening efforts and performance will be essential in order to improve preventive care for people with HIV.

## Data Availability

Due to patient data protection requirements the dataset supporting the findings of this study is not publicly available. Anonymized data are available from the authors upon reasonable request.

## Declarations

### Poster Presentation

Parts of these data has been presented at DÖAK 2025, Vienna: Menzel V., Esser S., Grüner E., Corominas A., Bogner J., Roider J., Seybold U., Anal cytology screening for people with HIV in a real-world setting – are we making a difference or just wasting time? 12th German-Austrian AIDS Conference DÖAK, Abstract 79, March 20–22 2025; Vienna, Austria. Presented by Viktoria Menzel.
